# Menopausal hormone therapy and risk of neuropsychiatric disease: a drug target Mendelian randomisation study

**DOI:** 10.1038/s44294-026-00130-1

**Published:** 2026-02-28

**Authors:** Louise S. Schindler, Dipender Gill, Hannah Oppenheimer, Claudia Barth, Ole A. Andreassen, Bogdan Draganski, Lars T. Westlye, Anya Topiwala, Ann-Marie G. de Lange

**Affiliations:** 1 Social, Genetic and Developmental Psychiatry Centre, King’s College London, London, UK; 2 Dept. of Psychiatry, University of Oxford, Oxford, UK; 3 Dept. of Epidemiology and Biostatistics, Imperial College London, London, UK; 4 Dept. of Psychology, University of Oslo, Oslo, Norway; 5 Division of Mental Health and Substance Abuse, Diakonhjemmet Hospital, Oslo, Norway; 6 Centre for Precision Psychiatry, Division of Mental Health and Addiction, Oslo University Hospital and Institute of Clinical Medicine, University of Oslo, Oslo, Norway; 7 KG Jebsen Centre for Neurodevelopmental Disorders, University of Oslo, Oslo, Norway; 8 Neurology Dept. and University Institute for Diagnostic and Interventional Neuroradiology, Inselspital, University Hospital Bern, University of Bern, Bern, Switzerland; 9 Nuffield Dept. of Population Health, Big Data Institute, University of Oxford, Oxford, UK; 10 School of Biological and Behavioural Sciences, Queen Mary University of London, London, UK

**Keywords:** Hormonal therapies, Reproductive biology

## Abstract

Evidence on whether menopausal hormone therapy (MHT) affects neurological or psychiatric disease is conflicting. As MHT acts by binding to oestrogen receptors (ER*α* and ER*β*), we used drug-target Mendelian randomisation (MR) to test whether perturbing these targets alters the risk of Alzheimer’s disease (AD), brain structure, depression, or anxiety. Genetic variants in the genes encoding these oestrogen receptors (*ESR1* and *ESR2*) that were associated with positive controls were leveraged as instrumental variables. In two-sample MR analyses using large genome-wide association studies, genetically proxied ER*α* and ER*β* perturbation showed no evidence of effect on AD or on cortical grey matter, hippocampal volume, or white matter hyperintensities. Genetically proxied ER*β* perturbation significantly increased risk for depression (*β* = −0.66, 95% CI [−0.99, −0.32], *p* = 0.002), but not anxiety. Our study highlights psychiatric considerations when targeting oestrogen receptors with MHT, but provides no evidence for either harmful or protective effects on AD risk.

## Introduction

Women are disproportionately affected by psychiatric and neurodegenerative disorders, including depression, anxiety, and Alzheimer’s disease (AD)^1–3^. One potential explanation for this female preponderance is an aetiological role of sex hormones such as oestrogens^4–7^. Oestradiol, the most abundant and potent oestrogen in the female body, exerts dynamic effects on brain morphology, neurochemistry, and function^8–12^, and is generally considered neuroprotective^13–16^. During the menopause transition, menopausal hormone therapy (MHT) is often prescribed to alleviate symptoms^17,18^ by partially replenishing sex hormones^19,20^. However, the effects of MHT use on mental health, AD risk, and markers of neurodegeneration are widely debated^4,21–25^, and the risks and benefits remain unclear.

MHT might help to mitigate neurological symptoms during the menopause transition^26–29^ with potential long-term implications for brain health^28,30^. However, findings from observational studies on MHT use are mixed. Some studies report an increased risk of AD or dementia^31–33^, and adverse effects on brain structure and neurodegenerative markers^34–36^. Other studies indicate a decreased AD risk^37–41^ and beneficial effects on brain health^42–44^, while some research finds no clinically relevant associations with neurological outcomes^45–47^. A meta-analysis of four randomised controlled trials (RCTs), based on data from the Women’s Health Initiative Memory Study (WHIMS), found an increased risk of dementia with MHT use compared to placebo^30^, though potential limitations of this dataset have been discussed^48^. The role of MHT in psychiatric disorders is also unclear. Some RCTs report a reduction in depressive symptoms with MHT use in menopausal females with depression^49,50^, as well as prevention of menopausal depression and anxiety^51–53^. Other RCTs reported no impact of MHT on depression and anxiety^27,54,55^, while some observational studies have shown an increased risk of depression in MHT users^56,57^.

Discrepancies in findings across studies may partially arise from reverse causation or confounding factors, particularly in observational studies. For example, individuals with severe menopausal symptoms including cognitive difficulties or mood changes^58^ may be more likely to be prescribed MHT^36,45^ or receive it following surgery (i.e., hysterectomy or oophorectomy^59,60^). Differences in factors such as MHT formulation, dosage, timing of onset, and duration of use are also likely to influence study results^4,7,30,36^. Furthermore, although RCTs are considered the gold standard in establishing causal effects^61^, their limited duration may preclude examination of long-term outcomes like AD^30,62^.

Mendelian randomisation (MR) is an epidemiological method that utilises genetics in a quasi-experimental approach to estimate causal effects^63^. Random allocation of genetic variants predicting a given phenotype at conception is analogous to random allocation to intervention in RCTs^64^. Genetic variants cannot be influenced by environmental processes, which minimises concerns about confounding and reverse causation^63^.

**Fig. 1:**
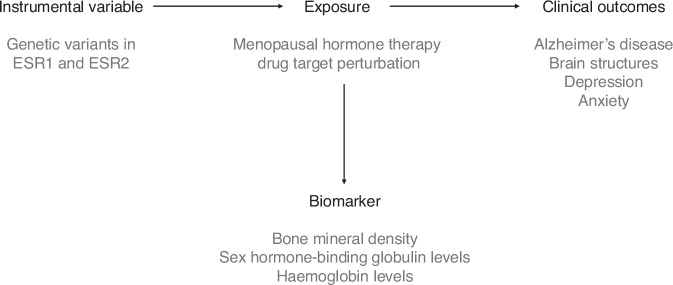
Principles of Mendelian Randomisation for studying drug effects. Genetic variants in *ESR1* and *ESR2*, which encode oestrogen receptors *α* and *β*, and that influence downstream biomarkers similar to the desired drug target effect (e.g., higher bone mineral density), are used as instrumental variables to proxy target perturbation. The causal effects of this genetically proxied perturbation on clinical outcomes (e.g., Alzheimer’s disease) are then estimated using Mendelian randomisation (MR).

MR can be extended to investigate the causal effects of intervening on drug target proteins^65–67^ (Fig. 1). This includes the two main oestrogen receptors (ER*α* and ER*β*); these proteins are encoded by genes *ESR1* and *ESR2*, respectively, and are targeted by oestrogen therapies such as MHT^13,68,69^. Genetic variants such as single-nucelotide polymorphisms (SNPs) that are located in the gene encoding the drug targets of interest (in this case, *ESR1* and *ESR2*) can be used to proxy the effects of pharmacological intervention at a specific receptor if they are associated with downstream effects similar to the desired drug response^65,70^.

These SNPs can be used as instrumental variables in MR analyses to study the effect of these drug target perturbations on psychiatric and neurological outcomes^65^. With increasing availability of large-scale genetic data^71,72^, such drug-target MR studies present unprecedented opportunities to assess these relationships while avoiding resource constraints of RCTs and potential confounding in observational studies^73^.

Here, we performed the first drug-target MR study examining the causal effects of MHT on AD risk, key brain structural outcomes, depression, and anxiety, all of which are more common in females compared to males. To do this, we used genetic variants that mimic the effects of MHT as instrumental variables. Specifically, we selected SNPs in the *ESR1* and *ESR2* genes that had been associated in genome-wide association studies (GWAS) with relevant biomarkers representing downstream effects of drugs targeting these receptors (bone mineral density, sex hormone-binding globulin (SHBG) levels, and haemoglobin levels) at genome-wide significance (*p* < 5 × 10^−8^). We tested associations of these variants with AD risk, cortical grey matter (GM) volume, hippocampal volume, white matter hyperintensity (WMH) volume, depression, and anxiety.

## Results

### Main findings

We identified three instruments to proxy pharmacological perturbation of the oestrogen receptors: seven SNPs in *ESR1* associated with bone mineral density, one SNP in *ESR1* associated with SHBG levels, and one SNP in *ESR2* associated with haemoglobin levels (Table 1).

**Table 1:** Main SNPs used to proxy ER*α* and ER*β* perturbation

Gene	Biomarker	SNP	EA	Beta	SE	*p*-value	Study	Sample size
*ESR1*	BMD	rs2504069	C	−0.042	0.002	2.2 × 10^−82^	Morris (2019)	426,824
		rs6905582	G	0.057	0.002	2.7 × 10^−92^	Morris (2019)	426,824
		rs2982573	T	−0.077	0.002	1.1 × 10^−305^	Morris (2019)	426,824
		rs2234693	T	−0.017	0.002	1.8 × 10^−12^	Morris (2019)	426,824
		rs10484920	A	0.039	0.004	1.2 × 10^−18^	Kim (2018)	394,929
		rs115192536	G	−0.053	0.004	6.4 × 10^−35^	Kim (2018)	394,929
		rs547908752	C	0.08	0.007	4.0 × 10^−27^	Kim (2018)	394,929
*ESR1*	SHBG	rs1738386	C	0.020	0.003	1.5 × 10^−9^	Haas (2022)	196,901
*ESR2*	HMG	rs1256061	T	−0.021	0.002	1.6 × 10^−23^	Oskarsson (2020)	684,122

Lead single-nucleotide polymorphisms (SNPs) in *ESR1* and *ESR2* selected for the instruments. All SNPs are intron variants. SNPs within each biomarker are uncorrelated. Summary statistics were obtained from Morris et al.^118^, Kim et al.^119^, Haas et al.^137^, and Oskarsson et al.^107^. *EA* effect allele, *SE* standard error, *BMD* bone mineral density, *SHBG* sex hormone-binding globulin, *HMG* haemoglobin.

**Fig. 2:**
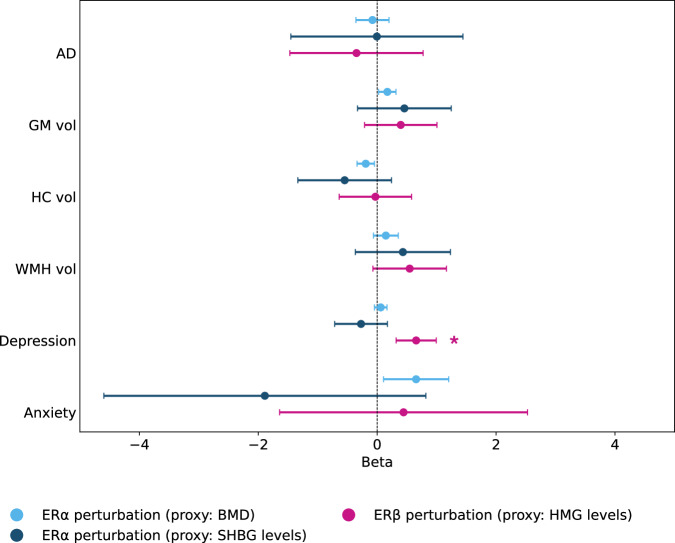
Two-sample Mendelian randomisation estimates for ER*α*/ER*β* perturbation. Forest plot showing beta estimates with 95% confidence intervals for genetically proxied perturbation of the oestrogen receptor *α* (ER*α*; proxied by bone mineral density [BMD] and sex hormone-binding globulin [SHBG]) and oestrogen receptor *β* (ER*β*; proxied by haemoglobin [HMG]) on Alzheimer’s disease (AD), grey matter volume (GM vol), hippocampal volume (HC vol), white matter hyperintensity volume (WMH vol), depression, and anxiety. For ER*β* associations, all estimated signs have been reversed for illustration. Only inverse-variance weighted (IVW) or Wald ratio estimates are shown. Findings significant at a false discovery rate of 5% are marked with an asterisk.

No significant associations were found between genetically predicted ER*α* and ER*β* perturbation and AD or WMH volume (Fig. 2, Tables 2, 3, and 4).

**Table 2:** Two-sample MR estimates for ER*α* perturbation proxied by bone mineral density

Outcome	Method	SNPs	Estimate	LCI	UCI	*p*	*p*_FDR_
AD	IVW	6	−0.079	−0.358	0.199	0.578	0.693
	Weighted median	6	−0.077	−0.400	0.245	0.638	
	Weighted mode	6	−0.077	−0.437	0.283	0.694	
	Simple mode	6	−0.128	−0.574	0.318	0.597	
	MR Egger	6	−0.021	−0.67	0.629	0.953	
Cortical GM volume	IVW	7	0.171	0.025	0.317	**0.021**	0.096
	Weighted median	7	0.163	−0.011	0.337	0.066	
	Weighted mode	7	0.176	−0.001	0.353	0.099	
	Simple mode	7	0.195	−0.068	0.458	0.196	
	MR Egger	7	0.112	−0.233	0.456	0.554	
HC volume	IVW	7	−0.192	−0.338	−0.046	**0.010**	0.089
	Weighted median	7	−0.165	−0.346	0.016	0.075	
	Weighted mode	7	−0.171	−0.35	0.008	0.109	
	Simple mode	7	−0.249	−0.546	0.047	0.150	
	MR Egger	7	−0.323	−0.667	0.022	0.126	
WMH volume	IVW	7	0.146	−0.064	0.356	0.172	0.391
	Weighted median	7	0.143	−0.029	0.315	0.104	
	Weighted mode	7	0.132	−0.055	0.318	0.216	
	Simple mode	7	0.196	−0.058	0.45	0.182	
	MR Egger	7	0.489	0.060	0.918	0.076	
Depression	IVW	6	0.060	−0.047	0.166	0.273	0.400
	Weighted median	6	0.055	−0.044	0.155	0.273	
	Weighted mode	6	0.047	−0.052	0.147	0.394	
	Simple mode	6	0.087	−0.086	0.259	0.371	
	MR Egger	6	0.106	−0.169	0.381	0.49	
Anxiety	IVW	6	0.655	0.107	1.203	**0.019**	0.096
	Weighted median	6	0.668	0.081	1.256	**0.026**	
	Weighted mode	6	0.686	0.008	1.364	0.104	
	Simple mode	6	0.251	−0.764	1.267	0.648	
	MR Egger	6	0.966	−0.300	2.233	0.209	

Effect sizes with 95% confidence intervals are shown for associations with Alzheimer’s disease (AD), cortical grey matter (GM) volume, hippocampal (HC) volume, white-matter hyperintensity (WMH) volume, depression, and anxiety using inverse-variance weighted (IVW), weighted median, weighted mode, simple mode, and MR Egger methods. Significant *p*-values (before or after FDR correction) are in bold. *ER* oestrogen receptor, *SNP* single nucleotide polymorphism, *LCI* lower confidence interval, *UCI* upper confidence interval.

**Table 3:** Two-sample MR estimates for ER*α* perturbation proxied by sex hormone-binding globulin levels

Outcome	Method	SNPs	Estimate	LCI	UCI	*p*	*p*_FDR_
AD	Wald ratio	1	−0.005	−1.452	1.442	0.995	0.995
Cortical GM volume	Wald ratio	1	0.457	−0.330	1.245	0.255	0.400
HC volume	Wald ratio	1	−0.547	−1.334	0.241	0.174	0.391
WMH volume	Wald ratio	1	0.433	−0.367	1.233	0.289	0.400
Depression	Wald ratio	1	−0.271	−0.714	0.172	0.230	0.400
Anxiety	Wald ratio	1	−1.889	−4.596	0.819	0.172	0.391

Effect sizes with 95% confidence intervals are shown for associations with Alzheimer’s disease (AD), cortical grey matter (GM) volume, hippocampal (HC) volume, white-matter hyperintensity (WMH) volume, depression, and anxiety using inverse-variance weighted (IVW), weighted median, weighted mode, simple mode, and MR Egger methods. Significant *p*-values (before or after FDR correction) are in bold. *ER* oestrogen receptor, *SNP* single nucleotide polymorphism, *LCI* lower confidence interval, *UCI* upper confidence interval.

**Table 4:** Two-sample MR estimates for ER*β* perturbation proxied by haemoglobin levels

Outcome	Method	SNPs	Estimate	LCI	UCI	*p*	*p*_FDR_
AD	Wald ratio	1	0.348	−0.773	1.468	0.543	0.693
Cortical GM volume	Wald ratio	1	−0.396	−1.006	0.213	0.203	0.400
HC volume	Wald ratio	1	0.030	−0.580	0.640	0.924	0.978
WMH volume	Wald ratio	1	−0.547	−1.166	0.072	0.084	0.301
Depression	Wald ratio	1	−0.656	−0.992	−0.319	**0.001**	**0.002**
Anxiety	Wald ratio	1	−0.444	−2.528	1.640	0.676	0.761

Effect sizes with 95% confidence intervals are shown for associations with Alzheimer’s disease (AD), cortical grey matter (GM) volume, hippocampal (HC) volume, white-matter hyperintensity (WMH) volume, depression, and anxiety using inverse-variance weighted (IVW), weighted median, weighted mode, simple mode, and MR Egger methods. Positive estimates indicate a negative relationship between ER*β* perturbation and the outcomes due to perturbation being proxied by lower haemoglobin levels. Significant *p*-values (before or after FDR correction) are in bold. *ER* oestrogen receptor, *SNP* single nucleotide polymorphism, *LCI* lower confidence interval, *UCI* upper confidence interval.

Genetically predicted ER*α* perturbation, proxied by bone mineral density, showed associations with higher cortical GM volume (IVW *β* = 0.171, 95% CI [0.025, 0.317], *p* = 0.021), as well as lower hippocampal volume (IVW *β* = −0.192, 95% CI [−0.338, −0.046], *p* = 0.010), though neither association remained significant after FDR correction (*p* = 0.160 and *p* = 0.148, respectively) (Table 2).

Genetically predicted ER*β* perturbation, proxied by haemoglobin levels, showed significant associations with higher risk of depression (Wald Ratio *β* = −0.656, 95% CI [−0.992, −0.319], *p* < 0.001, FDR-corrected *p* = 0.002) (Fig. 2, Table 4). Genetically predicted ER*α* perturbation, proxied by bone mineral density, showed associations with higher anxiety risk (IVW *β* = 0.655, 95% CI [0.107, 0.319], *p* = 0.019), but this did not remain significant after FDR correction (*p* = 0.160) (Table 2).

## Discussion

This MR study examined the effects of genetically predicted oestrogen receptor perturbation on neuropsychiatric outcomes and brain structure. In brief, we did not find significant effects on AD, brain structure, and anxiety, but genetically predicted ER*β* perturbation was significantly associated with a higher risk of depression.

There was no evidence for significant associations between genetically proxied perturbation of ER*α* or ER*β* and AD, indicating that activity of these receptors, which are targeted by MHT, does not influence AD risk. This finding contrasts with WHIMS^74^, which reported a doubled risk of developing dementia among postmenopausal women age 65 and older, though primarily for orally administered conjugated equine oestrogen therapy with progesterone^30^. Other trials enroling younger females or administering MHT closer to menopause have demonstrated mostly neutral effects of MHT on cognition^35,52,75,76^. This discrepancy could potentially be explained by the critical window hypothesis, which suggests that the benefits of MHT are contingent on early initiation^77–79^. Although MR estimates can provide insights into lifelong effects of genetic variants, they do not equate to the impacts of pharmacological interventions initiated at specific times^65^. Therefore, despite the lack of evidence for a link between oestrogen receptor perturbation and AD, potential risks and benefits associated with the timing of MHT initiation remain possible.

Furthermore, no evidence was found for associations between genetically predicted ER*α* or ER*β* perturbation and brain structural outcomes, indicating that drugs targeting these receptors do not seem to impact cortical GM volume, hippocampal volume, or WMH volume. Although ER*α* was associated with higher cortical GM volume and lower hippocampal volume, these findings did not remain significant after FDR correction. In a previous RCT study, recently postmenopausal females receiving conjugated equine oestrogens for four years had greater ventricular volume increases compared to placebo^35^, but the increase was not different from placebo three years after discontinuation^76^. In a separate trial, there were no significant differences in total hippocampal volume between multiple doses of oestradiol and placebo following short-term administration, though higher bilateral posterior hippocampal volume was increased after three months at the highest dose^80^. Overall, the reported impact of MHT on GM changes ranges from volume increases to decreases or null-effects (for a full review, see ref. ^6^), and comparability of studies is complicated by confounders such as age, mental health status, formulation, and duration of MHT use^6,7^.

While relationships between MHT use and brain health might depend on formulation, dosage, timing of onset, and duration of use, the previously published GWAS, whose data we relied on, may also be subject to the healthy volunteer effect^81^ and survivor bias^82^. These biases, which result from the tendency of healthier individuals to participate and the exclusion of those who have died or experienced severe illness, may affect the generalisability of our findings.

Our analyses used GWAS summary statistics for AD, brain volumes, and depression derived from large meta-analyses. Phenotype definitions may differ across contributing cohorts, particularly for clinical and psychiatric outcomes, and this heterogeneity could introduce measurement error and attenuate causal estimates^83^. We restricted our analyses to European-ancestry samples to reduce confounding by population stratification, which improves internal validity. However, we primarily relied on variant-biomarker and variant-outcome associations reported in both males and females, mainly due to the low availability of sex-stratified GWAS. While our results are therefore generalisable to both sexes (i.e., no associations between oestrogen receptor perturbation and AD risk in both males and females), mixed-sex summary statistics may limit the precision of female-specific estimates. While these limitations highlight the need for future GWAS with harmonised outcome definitions and sex-stratified analyses, our cis-MR design using variants proximal to drug targets provides a conservative and biologically informed approach to causal inference.

Genetically predicted ER*β* perturbation, proxied by haemoglobin levels, was significantly associated with higher depression risk, supporting the role of oestrogens in mental health. Oestrogens are known to influence mood regulation through various mechanisms in the brain, such as modulating neurotransmitter systems and neuroplasticity^84–86^, but the role of both endogenous and exogenous oestrogens in mood disorders is complicated and multifaceted. For instance, studies suggest that sensitivity to fluctuations in sex hormone levels may drive depressive symptoms in premenstrual dysphoric disorder (PMDD) or perimenopause^87–89^. Hormonal contraceptive use has been associated with increased risk of depression in observational studies^90–93^, especially in adolescents^92^. In contrast, a recent network meta-analysis including 14 RCTs concluded that hormonal contraceptive use did not lead to increased depressive symptoms in adult females^94^. Similarly, MHT use has been shown to be generally neutral^54,55^ or even protective^49–52^ for depressive symptoms in observational studies and RCTs.

Our study indicated an increased risk of depression associated with ER*β* perturbation; however, as noted above, while our methods can provide evidence of the presence and direction of causal effects, they are not equivalent to pharmacological intervention^65^. Additionally, MR studies work under the assumption of constant genetic exposure effects over a lifetime, which may not capture the reality of time-varying effects^95^. As the relationship between oestradiol and psychiatric outcomes may change across different life stages^84^, future MR studies could employ methods such as multivariable MR to examine time-varying effects^96^, though this would require several GWAS on relevant traits across different age groups or timepoints in an individual’s life. Accordingly, we could not assess age- or menopause-specific genetic effects in this study because suitable age-or menopause-stratified GWAS were unavailable.

We did not find evidence supporting a role of ER*α* in the risk of depression. ER*α* perturbation was associated with higher anxiety risk, though this was not significant following FDR correction. Both receptor subtypes are located in brain regions associated with cognitive function and emotion^97,98^, but there is higher expression of ER*β* in the thalamus and hippocampus^99^, areas involved in mood disorders^100–102^, which may explain our findings. Following future research confirming a differential role of ER*α* and ER*β* in depression, oestrogen receptor modulators that selectively act on ER*α* could be explored to avoid inducing depressive symptoms with hormone therapy use.

There are some limitations in the use of haemoglobin as a proxy for ER*β* perturbation. While higher oestrogen has been correlated with lower haemoglobin levels^103–106^, we could not identify any RCTs that linked use of exogenous oestrogens to lower haemoglobin, making this proxy less robustly validated than, for example, bone mineral density for ER*α* perturbation. Additionally, as about two-thirds of the participants in the haemoglobin level GWAS^107^ were from the UK Biobank, we expected substantial sample overlap between the phenotype and outcome samples, which could introduce population-specific effects affecting the robustness of the results. Finally, using haemoglobin to proxy ER*β* assumes it does not influence depression independently of ER*β* activity (exclusion restriction). However, plausible pathways exist from low haemoglobin to fatigue and depressive symptoms. Our follow-up standard MR analysis (Supplementary Note 1) showed an association between haemoglobin and depression by IVW, though not across sensitivity methods, suggesting potential partial violation of this assumption. We therefore interpret the ER*β* perturbation and depression result cautiously, emphasising that further research with alternative ER*β* proxies is needed to confirm this finding.

This study represents an important initial step in using drug target MR studies to identify risks and benefits associated with MHT use. Although these studies cannot fully substitute for the precision and specific temporal dynamics captured in clinical pharmacological trials, this method can validate drug targets, identify relevant side-effects and outcomes, and aid in drug repurposing by identifying new therapeutic uses for existing drugs, without the high cost and long duration associated with RCTs^65,66^. Triangulation of findings across these different methodological approaches is essential for a robust understanding of the causal effects of MHT.

Although our current results should be interpreted in the context of the discussed limitations, they provide a foundation for future research elucidating biological pathways and evaluating long-term safety and efficacy of MHT use to optimise women’s healthcare oucomes. For example, well-powered sex-stratified GWAS are mostly lacking, and in addition to AD or psychiatric outcomes, GWAS on depression subtypes such as postpartum depression or PMDD would improve our understanding of female-specific findings related to hormonal treatments. Future analyses could also aim to examine the contribution of progesterone receptor perturbation on neuropsychiatric and brain structural outcomes. Exogenous oestradiol, via MHT or hormonal contraception, is commonly combined with progesterone to balance its effects^108^, and various formulations might differentially affect risk of mental disorders^92,109^ as well as AD^24,30,110^. At the time of the current study, genetic variants within the PGR gene had not been associated with relevant biomarkers representing downstream drug effects, and we were thus unable to explore its role in these neuropsychiatric outcomes. Given the variety of MHT combinations, it is vital to conduct future studies that can provide a basis for clarifying the causal effects of these on health outcomes.

In conclusion, this study found no evidence that genetically predicted ER perturbation, as targeted by MHT, significantly affects AD risk or associated brain structural measures, including cortical GM volume, hippocampal volume, and WMH volume. However, ER*β* perturbation may be associated with higher depression risk, indicating a potential causal role of oestrogen in mood regulation. Future drug target MR studies can complement observational studies and RCTs by offering critical insights into the causal effects of MHT on various health outcomes. This approach is essential for clarifying inconsistent findings and guiding pharmaceutical research, ultimately optimising patient care and ensuring long-term health benefits for women.

## Methods

### Study design and instrument selection

Figure 1 illustrates the principles of MR studies used to investigate drug effects. By utilising genetic variants that proxy receptor activity, this approach can help us understand whether the binding of oestrogens to oestrogen receptors, the primary aim of MHT, has a direct impact on AD risk, brain structural outcomes, and mental health.

To proxy oestrogen receptor perturbation, we gathered all reported associations between SNPs located in the *ESR1* and *ESR2* genes and various biomarkers, based on the NHGRI-EBI GWAS Catalog (www.ebi.ac.uk/gwas/home)^111^. We selected only SNPs located within 200kb of the gene start and end (genome build 38p14; location for *ESR1* = chromosome 6, 151,656,691 - 152,129,619, and for *ESR2* = chromosome 14, 64,084,232 - 64,338,112) and associated with biomarkers at genome-wide significance (*p* < 5 × 10^−8^). We removed any associations observed in male-only samples to ensure we captured female-specific effects, or non-European samples, given that different genetic ancestries can influence allele frequencies and linkage disequilibrium (LD) patterns^112^, and the lack of available ancestry-specific outcome GWAS. Table 5 provides an overview of exclusions for instrumental variable selection.

**Table 5:** Exclusions applied to SNP associations for *ESR1* and *ESR2*

	*ESR1*	*ESR2*
Number of reported SNP associations	293	102
Number above *p* < 5 × 10^−8^	34	9
Number ± 200kb outside of gene region	0	0
Number of male-only samples	13	1
Number of non-European or mixed ancestry samples	97	22
Number of SNP associations following biomarker selections	35	4

Counts of *ESR1* and *ESR2* SNP associations from the NHGRI-EBI GWAS Catalog that were excluded at each step: not meeting genome-wide significance (*p* < 5 × 10^−8^), ±200 kb outside of the gene region, male-only samples, and non-European or mixed-ancestry samples. The final row reports SNPs retained after biomarker-based selection for instrument construction. *SNP* single-nucleotide polymorphism, *GWAS* genome-wide association study, *kb* kilobases.

Biomarkers were chosen based on their biological plausibility to mimic drug target protein effects. For *ESR1*, we selected “bone mineral density" and “sex hormone-binding globulin (SHBG) levels". Increased bone mineral density or reduced fracture risk has been observed in participants taking MHT compared to placebo in several RCTs^113–116^. SHBG concentrations are significantly higher during use of hormonal contraception (which targets ER*α*) in a dose-response manner, as highlighted by a meta-analysis of experimental studies^117^. As there were two plausible biomarkers available, we selected both, as consistent results across these would strengthen our results. For *ESR2*, we chose the biomarker “haemoglobin levels". Females have lower haemoglobin levels compared to males^103^, with times marked by increased levels of oestrogens (i.e., pregnancy) resulting in reductions in haemoglobin^104^. In transgender participants receiving oestradiol therapy, haemoglobin decreased significantly as oestradiol levels increased^105,106^.

Following biomarker selections, there were 30 SNP associations for bone mineral density, 5 for SHBG levels, and 4 for haemoglobin levels (Table 5); some SNPs were associated with a biomarker in several studies. Where SNPs associated with the same biomarker were in LD (r2 < 0.1), we selected a main SNP and its associated statistics based firstly on whether it had been identified in more than one sample, secondly, the sample size of the study reporting the association, and thirdly, the availability of summary statistics. Where the same SNP association was reported in several studies, summary statistics were obtained from the study with the largest sample size. All SNPs not in LD with any other SNPs were also selected as main SNPs. Summary statistics for the main SNPs were downloaded from the GWAS Catalog or retrieved manually from the publication. Supplementary Tables 1–3 provide the full list of studies reporting associations for SNPs in *ESR1* and *ESR2* with the biomarkers, including information on LD and availability of summary statistics. Table 1 displays the final selection of SNP associations comprising the three instruments reflecting ER*α* and ER*β* perturbation. We obtained summary statistics from the largest available study for each biomarker rather than meta-analysing across multiple GWAS due to substantial sample overlap between studies (e.g., UK Biobank participants appear in both ref. ^118^ and ref. ^119^), differing measurement platforms and phenotype definitions across cohorts, and because most additional studies were considerably smaller and would contribute minimal power while introducing heterogeneity.

### Outcome selection

Our primary outcomes of interest were AD, depression, and anxiety, given the female preponderance of these conditions and the conflicting evidence surrounding associations with exogenous oestrogens^1,4^. We further wanted to investigate associations with relevant brain structural outcomes; GM and hippocampal volume changes have been closely associated with the onset and progression of dementia^120^, as well as depression^121,122^ and anxiety^123,124^. Finally, as recent evidence highlights menopause as a risk factor for WMHs^125,126^, which have also been associated with increased risk of AD^127,128^, WMH volume was included as an outcome.

Variant-outcome associations were derived from large previously published GWAS. For AD, we selected a meta-analysis of GWAS on clinically diagnosed late-onset AD with 94,437 individuals^129^. Genetic associations with brain structural outcomes (cortical GM, hippocampal, and WMH volume), were obtained from the largest GWAS of brain imaging phenotypes from the UK Biobank with 22,138 individuals^130^. For depression, we selected a meta-analysis of three GWAS on a spectrum of depression phenotypes with 807,553 individuals (246,363 cases and 561,190 controls)^131^, and for anxiety, we chose a meta-analysis of nine GWAS on anxiety disorders with 17,310 individuals^132^. All genetic associations were based on GWAS of European ancestry samples. Variant-outcome associations for the genetic instruments are provided in Supplementary Tables 4–9.

### Mendelian randomisation

Two-sample MR analyses were used to obtain estimates for the association between genetically predicted oestrogen receptor perturbation, proxied by relevant biomarkers, and AD, cortical GM volume, hippocampal volume, WMH volume, depression, and anxiety. Analyses were conducted using the *TwoSampleMR* (version 0.5.6) package in R (version 4.2.1). Variants were harmonised between datasets, ensuring that the associations between SNPs and exposure and between SNPs and the outcome reflected the same allele. For instruments with more than two variants, the Inverse Variance Weighted (IVW)^70^ method was performed as the primary approach, which regresses the effect sizes of the variant-biomarker associations against the effect sizes of the variant-outcome associations. Several other methods, such as weighted median^133^, weighted and simple mode^134^, and MR Egger^135^ were performed to assess the robustness of results, as broadly consistent results across these methods strengthen the causal inference. For instruments with a single variant, the Wald ratio method was employed. To adjust for multiple testing, false discovery rate (FDR; 5%)^136^ corrected p-values were calculated across all instances of IVW and Wald ratio methods. To assess potential violation of the exclusion restriction where a proxy biomarker might itself causally influence an outcome (e.g., haemoglobin - depression), we conducted standard follow-up MR analyses of the biomarker on the outcome for any significant results (see Supplementary Note 1).

### Ethical considerations

This study used publicly available summary statistics from previously published GWAS. No new data involving human participants were collected or analysed by the authors. Ethical approval for all contributing studies was obtained from the respective institutional review boards or ethics committees, and all participants provided written informed consent in accordance with the Declaration of Helsinki. Studies utilising UK Biobank data^107,118,119,130,137^ operated under ethical approval from the North West Multi-Centre Research Ethics Committee. For ref. ^129^, all contributing cohorts obtained approval from local Institutional Review Boards; the AGES Reykjavik Study was approved by the Icelandic National Bioethics Committee (VSN 00-063), the Icelandic Data Protection Authority, and the U.S. NIA IRB; and CHARGE consortium studies received approval from respective local IRBs. For ref. ^107^, the Icelandic component received approval from the Icelandic National Bioethics Committee (VSNb2015010033-03.12). For ref. ^131^, UK Biobank data were used under approval from the NHS National Research Ethics Service (11/NW/0382), with additional approvals for Generation Scotland (NHS Tayside Committee on Research Ethics, Ref 15/ES/0040), BiDirect and Münster cohorts (Ethics Committee of the University of Münster and Westphalian Chamber of Physicians), 23andMe (external AAHRPP-accredited institutional review board), and 35 Psychiatric Genomics Consortium cohorts as detailed in the original publication. For ref. ^132^, contributing cohorts received approvals from MGS (institutional review boards at participating US institutions), PsyCoLaus (local institutional review board, Switzerland), SHIP (Ethics Committee of the University of Greifswald, Germany), QIMR (Human Research Ethics Council of QIMR, Australia), TRAILS (Dutch Central Committee on Research Involving Human Subjects), Rotterdam Study (appropriate ethical approvals, Netherlands), and NESDA/NTR (Central Ethics Committee on Research Involving Human Subjects of the VU University Medical Center, Amsterdam). To the best of our knowledge, all other contributing studies complied with relevant ethical regulations and obtained informed consent, as detailed in the original publications.

## Supplementary information

Supplementary Information

## Data Availability

This study used publicly available summary statistics from previously published GWAS. No new data was generated. The data is available in the NHGRI-EBI Catalog of human genome-wide association studies or in the summary statistics from published GWAS. The analysis script is publicly available on the GitHub repository: https://github.com/louisesophieschindler/Drug-Target-Mendelian-Randomisation/tree/main.
