# Supplementary material for: Menopausal hormone therapy and risk of neuropsychiatric disease: a drug target Mendelian randomisation study
Source: NPJ Womens Health. 2026 Feb 28;4(1):10. doi: 10.1038/s44294-026-00130-1 (PMC12949953; doi:10.1038/s44294-026-00130-1)
Supplement: Supplementary file 1 — Supplementary Information [file 44294_2026_130_MOESM1_ESM.pdf]

Supplementary Information

Supplementary Tables

Supplementary Tables 1, 2, and 3 show the full list of single-nucleotide polymorphisms (SNPs) associated with bone mineral density, sex hormone-binding globulin (SHBG) levels, and haemoglobin levels, located in genes *ESR1* and *ESR2*. Information on linkage disequilibrium (LD) and availability of summary statistics is also reported. Main SNPs used in the analysis are marked in bold and reported in main manuscript Section 2.1.

Supplementary Table 1: ***ESR1* SNP associations with bone mineral density.** All NHGRI-EBI genome-wide association study (GWAS) Catalog reports of single-nucleotide polymorphism (SNP) associations in *ESR1* for the biomarker bone mineral density (BMD). Where SNPs were in linkage disequilibrium (LD) ($r^2 < 0.1$), the main SNP was selected based on 1) whether it had been identified in more than one study, and 2) the sample size of the study. Where the same SNP association was reported in several studies, summary statistics were obtained from the largest study with available summary statistics. Main SNPs used in the analyses are marked in bold.

SNP	Biomarker	Study	Sample Size	LD	Summary statistics
**rs2504069**	**Heel BMD**	**Morris (2018)**	**426,824**	**Main SNP**	**Available**
	Heel BMD	Kim (2018)	394,929		
	Heel BMD	Kemp (2017)	142,487		
rs1890010	Heel BMD	Kim (2018)	394,929	In LD (r^2=1.0).	
rs1999805	BMD (spine)	Styrkarsdottir (2008)	5,861	In LD (r^2=0.20).	
rs2504063	BMD (spine)	Rivadeneira (2009)	19,195	In LD (r^2=0.20).	
rs2982552	Heel BMD	Kim (2018)	394,929	In LD (r^2=0.16).	
rs3020304	Heel BMD	Kichaev (2018)	446,000	In LD (r^2=0.15).	
**rs2982573**	**Heel BMD**	**Morris (2018)**	**426,824**	**Main SNP**	**Available**
	Heel BMD	Kim (2018)	394,929		
	Ultradistal forearm BMD	Surakka (2020)	21,907		
rs2941741	Heel BMD	Kim (2018)	394,929	In LD (r^2=0.99).	
	Heel BMD	Kemp (2017)	142,487	In LD (r^2=0.99).	
	Heel BMD	Kim (2018)	394,929	In LD (r^2=0.99).	
rs2941740	BMD (hip)	Rivadeneira (2009)	19,195	In LD (r^2=1.0).	
rs3020331	Heel BMD	Wang (2019)	197,261	In LD (r^2=0.94).	
rs3020332	Heel BMD	Kichaev (2018)	∼446,000	In LD (r^2=0.89).	
rs851990	Heel BMD	Kichaev (2018)	∼446,000	In LD (r^2=0.11).	
**rs6905582**	**Heel BMD**	**Morris (2018)**	**426,824**	**Main SNP**	**Available**
	Heel BMD	Kim (2018)	394,929		
**rs2234693**	**Heel BMD**	**Morris (2018)**	**426,824**	**Main SNP**	**Available**
	Heel BMD	Kim (2018)	394,929		
**rs10484920**	**Heel BMD**	**Kim (2018)**	**394,929**	**Main SNP**	**Available**
**rs115192536**	**Heel BMD**	**Kim (2018)**	**394,929**	**Main SNP**	**Available**
**rs547908752**	**Heel BMD**	**Kim (2018)**	**394,929**	**Main SNP**	**Available**
rs1884051	Heel BMD	Kichaev (2018)	∼446,000	Main SNP	Not available, removed

Supplementary Table 2: ***ESR1* SNP associations with sex hormone-binding globulin.** All NHGRI-EBI genome-wide association study (GWAS) Catalog reports of single-nucleotide polymorphism (SNP) associations in *ESR1* for the biomarker sex hormone-binding globulin (SHBG). Where SNPs were in linkage disequilibrium (LD) ($r^2 < 0.1$), the main SNP was selected based on 1) whether it had been identified in more than one study, and 2) the sample size of the study. Where the same SNP association was reported in several studies, summary statistics were obtained from the largest study with available summary statistics. Main SNPs used in the analyses are marked in bold.

SNP	Biomarker	Study	Sample size	LD	Summary statistics
**rs1738386**	**SHBG levels**	**Haas (2022)**	**196,901**	**Main SNP**	**Available**
	SHBG levels	Haas (2022)	43,477		
	SHBG levels	Ruth (2020)	189,473		
	SHBG levels adjusted for BMI	Ruth (2020)	188,908		
rs1293953	SHBG	Leinonen (2023)	188,443	In LD (r^2=0.66).	

Supplementary Table 3: ***ESR2* SNP associations with haemoglobin.** All NHGRI-EBI genome-wide association study (GWAS) Catalog reports of single-nucleotide polymorphism (SNP) associations in *ESR2* for the biomarker haemoglobin. Where SNPs were in linkage disequilibrium (LD) ($r^2 < 0.1$), the main SNP was selected based on 1) whether it had been identified in more than one study, and 2) the sample size of the study. Where the same SNP association was reported in several studies, summary statistics were obtained from the largest study with available summary statistics. Main SNPs used in the analyses are marked in bold.

SNP	Biomarker	Study	Sample size	LD	Summary statistics
**rs1256061**	**Haemoglobin levels**	**Oskarsson (2020)**	**684,122**	**Main SNP**	**Available**
	Haemoglobin concentration	Chen (2020)	563,946		
	Haemoglobin	Vuckovic (2020)	408,112		
	Haemoglobin concentration	Astle (2016)	172,925		

Supplementary Tables 4, 5, 6, 7, 8, and 9 show the outcome genome-wide association study (GWAS) associations for genetic instruments used in the Mendelian randomisation (MR) analyses of Alzheimer's disease (AD), grey matter (GM) volume, hippocampal (HC) volume, white matter hyperintensity (WMH) volume, depression, and anxiety, respectively.

Supplementary Table 4: **Outcome GWAS associations for genetic instruments used in MR analysis of Alzheimer's disease.** Summary statistics from [1]. Listed are the effect allele (EA), effect estimate, standard error (SE), and p-value for each instrument single nucleotide polymorphism (SNP) in *ESR1* and *ESR2* across biomarkers: bone mineral density (BMD), sex hormone–binding globulin (SHBG), and haemoglobin (HMG). GWAS = genome-wide association study; MR = Mendelian randomisation.

Gene	Biomarker	SNP	EA	Beta	SE	p-value
ESR1	BMD	rs2504069	T	-0.0047	0.0159	0.7667
		rs6905582	A	-0.0032	0.0199	0.8740
		rs2982573	T	0.0055	0.0145	0.7033
		rs2234693	T	0.0026	0.0143	0.8559
		rs10484920	A	-0.0158	0.0314	0.6159
		rs115192536	A	-0.0118	0.0290	0.6833
		rs547908752	–	–	–	–
ESR1	SHBG	rs1738386	T	0.0001	0.0147	0.9958
ESR2	HMG	rs1256061	T	-0.0087	0.0143	0.5405

Supplementary Table 5: **Outcome GWAS associations for genetic instruments used in MR analysis of cortical GM volume.** Summary statistics from [2]. Listed are the effect allele (EA), effect estimate, standard error (SE), and *p*-value for each instrument single nucleotide polymorphism (SNP) in *ESR1* and *ESR2* across biomarkers: bone mineral density (BMD), sex hormone–binding globulin (SHBG), and haemoglobin (HMG). GWAS = genome-wide association study; MR = Mendelian randomisation.

Gene	Biomarker	SNP	EA	Beta	SE	*p*-value
ESR1	BMD	rs2504069	T	0.0005	0.0085	0.9520
		rs6905582	A	-0.0143	0.0104	0.1697
		rs2982573	C	0.0125	0.0078	0.1111
		rs2234693	C	0.0081	0.0078	0.2977
		rs10484920	G	-0.0064	0.0169	0.7039
		rs115192536	A	0.0139	0.0163	0.3934
		rs547908752	T	-0.0139	0.0270	0.6069
ESR1	SHBG	rs1738386	C	0.0091	0.0080	0.2548
ESR2	HMG	rs1256061	T	0.0099	0.0078	0.2025

Supplementary Table 6: **Outcome GWAS associations for genetic instruments used in MR analysis of hippocampal (HC) volume.** Summary statistics from [2]. Listed are the effect allele (EA), effect estimate, standard error (SE), and *p*-value for each instrument single nucleotide polymorphism (SNP) in *ESR1* and *ESR2* across biomarkers: bone mineral density (BMD), sex hormone–binding globulin (SHBG), and haemoglobin (HMG). GWAS = genome-wide association study; MR = Mendelian randomisation.

Gene	Biomarker	SNP	EA	Beta	SE	*p*-value
ESR1	BMD	rs2504069	T	-0.0122	0.0085	0.1508
		rs6905582	A	0.0229	0.0104	0.0276
		rs2982573	C	-0.0090	0.0078	0.2503
		rs2234693	C	0.0070	0.0078	0.3734
		rs10484920	G	0.0008	0.0169	0.9599
		rs115192536	A	-0.0105	0.0163	0.5181
		rs547908752	T	0.0354	0.0270	0.1886
ESR1	SHBG	rs1738386	C	-0.0109	0.0080	0.1737
ESR2	HMG	rs1256061	T	-0.0007	0.0078	0.9238

Supplementary Table 7: **Outcome GWAS associations for genetic instruments used in MR analysis of white matter hyperintensity (WMH) volume.** Summary statistics from [2]. Listed are the effect allele (EA), effect estimate, standard error (SE), and *p*-value for each instrument single nucleotide polymorphism (SNP) in *ESR1* and *ESR2* across biomarkers: bone mineral density (BMD), sex hormone–binding globulin (SHBG), and haemoglobin (HMG). GWAS = genome-wide association study; MR = Mendelian randomisation.

Gene	Biomarker	SNP	EA	Beta	SE	*p*-value
ESR1	BMD	rs2504069	T	0.0043	0.0086	0.6177
		rs6905582	A	-0.0153	0.0106	0.1484
		rs2982573	C	0.0079	0.0080	0.3191
		rs2234693	C	-0.0205	0.0079	0.0100
		rs10484920	G	-0.0251	0.0172	0.1452
		rs115192536	A	0.0286	0.0166	0.0843
		rs547908752	T	-0.0212	0.0274	0.4385
ESR1	SHBG	rs1738386	C	0.0086	0.0081	0.2891
ESR2	HMG	rs1256061	T	0.0137	0.0079	0.0835

Supplementary Table 8: **Outcome GWAS associations for genetic instruments used in MR analysis of depression.** Summary statistics from [3]. Listed are the effect allele (EA), effect estimate, standard error (SE), and *p*-value for each instrument single nucleotide polymorphism (SNP) in *ESR1* and *ESR2* across biomarkers: bone mineral density (BMD), sex hormone–binding globulin (SHBG), and haemoglobin (HMG). GWAS = genome-wide association study; MR = Mendelian randomisation.

Gene	Biomarker	SNP	EA	Beta	SE	*p*-value
ESR1	BMD	rs2504069	T	0.0053	0.0048	0.2697
		rs6905582	A	0.0046	0.0058	0.4282
		rs2982573	T	-0.0040	0.0043	0.3576
		rs2234693	T	0.0043	0.0043	0.3171
		rs10484920	A	0.0147	0.0092	0.1111
		rs115192536	A	0.0177	0.0088	0.0433
		rs547908752	–	–	–	–
ESR1	SHBG	rs1738386	T	0.0054	0.0045	0.2267
ESR2	HMG	rs1256061	T	0.0164	0.0043	0.0001

Supplementary Table 9: **Outcome GWAS associations for genetic instruments used in MR analysis of anxiety.** Summary statistics from [4]. Listed are the effect allele (EA), effect estimate, standard error (SE), and p-value for each instrument single nucleotide polymorphism (SNP) in *ESR1* and *ESR2* across biomarkers: bone mineral density (BMD), sex hormone–binding globulin (SHBG), and haemoglobin (HMG). GWAS = genome-wide association study; MR = Mendelian randomisation.

Gene	Biomarker	SNP	EA	Beta	SE	p-value
ESR1	BMD	rs2504069	C	-0.0652	0.0305	0.03262
		rs6905582	G	0.0277	0.0379	0.4643
		rs2982573	C	0.0548	0.0287	0.05619
		rs2234693	C	-0.0076	0.0275	0.7818
		rs10484920	G	0.0471	0.0573	0.411
		rs115192536	G	0.0030	0.0666	0.9635
		rs547908752	–	–	–	–
ESR1	SHBG	rs1738386	C	-0.0376	0.0275	0.1712
ESR2	HMG	rs1256061	G	-0.0111	0.0266	0.6772

Supplementary Note 1: Follow-up Analyses

MR analysis of haemoglobin levels and depression

Genetically-predicted haemoglobin levels were ascertained using an instrument composed of 659 variants associated at genome-wide significance ($p < 5 \times 10^{-8}$) with haemoglobin levels in the GWAS utilised for main analyses [5]. Genetic associations with depression were obtained from the same GWAS utilised for main analyses [3]. Two-sample MR analyses were used to obtain estimates for the association between genetically predicted haemoglobin levels and depression. Analyses were conducted as described in main manuscript section 4.3.

MR results for haemoglobin levels and depression

There was a significant association between genetically predicted haemoglobin levels and depression (IVW β = 0.061, 95% CI = [-0.013 - 0.073], $p < 0.001$), but this was not robust across sensitivity methods (MR Egger β = 0.030, 95% CI = [-0.013 - 0.073], p = 0.178; Weighted Median β = 0.010, 95% CI = [-0.014 - 0.034], p = 0.404; Simple Mode β = -0.030, 95% CI = [-0.081 - 0.021], p = 0.253; Weighted Mode β = -0.057, 95% CI = [-0.078 - 0.036], p = 0.611).

Supplementary References

[1] Kunkle, B. W. *et al.* Genetic meta-analysis of diagnosed Alzheimer's disease identifies new risk loci and implicates Aβ, tau, immunity and lipid processing. *Nat. Genet.* **51**, 414–430 (2019).

[2] Smith, S. M. *et al.* An expanded set of genome-wide association studies of brain imaging phenotypes in UK Biobank. *Nat. Neurosci.* **24**, 737–745 (2021).

[3] Howard, D. M. *et al.* Genome-wide meta-analysis of depression identifies 102 independent variants and highlights the importance of the prefrontal brain regions. *Nat. Neurosci.* **22**, 343–352 (2019).

[4] Otowa, T. *et al.* Meta-analysis of genome-wide association studies of anxiety disorders. *Mol. Psychiatry* **21**, 1391–1399 (2016).

[5] Oskarsson, G. R. *et al.* Predicted loss and gain of function mutations in ACO1 are associated with erythropoiesis. *Commun. Biol.* **3**, 189; 10.1038/s42003–020–0921–5 (2020).
